# Control Model for Dampening Hand Vibrations Using Information of Internal and External Coordinates

**DOI:** 10.1371/journal.pone.0125464

**Published:** 2015-04-13

**Authors:** Shunta Togo, Takahiro Kagawa, Yoji Uno

**Affiliations:** 1 Cognitive Mechanisms Laboratories, Advanced Telecommunications Research Institute International, Kyoto, Japan; 2 Japan Society for the Promotion of Science, Tokyo, Japan; 3 Graduate School of Engineering, Nagoya University, Nagoya, Japan; Duke University, UNITED STATES

## Abstract

In the present study, we investigate a control mechanism that dampens hand vibrations. Here, we propose a control method with two components to suppress hand vibrations. The first is a passive suppression method that lowers the joint stiffness to passively dampen the hand vibrations. The second is an active suppression method that adjusts an equilibrium point based on skyhook control to actively dampen the hand vibrations. In a simulation experiment, we applied these two methods to dampen hand vibrations during the shoulder’s horizontal oscillation. We also conducted a measurement experiment wherein a subject’s shoulder was sinusoidally oscillated by a platform that generated horizontal oscillations. The results of the measurement experiments showed that the jerk of each part of the arm in a task using a cup filled with water was smaller than the shoulder jerk and that in a task with a cup filled with stones was larger than the shoulder jerk. Moreover, the amplitude of the hand trajectory in both horizontal and vertical directions was smaller in a task using a cup filled with water than in a task using a cup filled with stones. The results of the measurement experiments were accurately reproduced by the active suppression method based on skyhook control. These results suggest that humans dampen hand vibrations by controlling the equilibrium point through the information of the external workspace and the internal body state rather than by lowering joint stiffness only by using internal information.

## Introduction

The human body has many degrees of freedom (DOFs) and can perform daily movements by dexterously coordinating redundant body parts without requiring special attention. Investigating a control mechanism of dexterous human movement supports the understanding of the role played by the human central nervous system (CNS) in motor control. We investigated human daily and dexterous movements that we previously described as “carrying a cup filled with water without spilling it” [1]. In our earlier study, we considered a hand vibration as a hand jerk (the rate of change of acceleration), and subjects reduced the hand jerk and maintained a constant cup angle by coordinating their multi-joints to carry a cup filled with water. However, the control mechanism that dampens hand vibrations, and thus reduces the hand jerks, remains unclear. This study investigates the human control mechanism that dampens hand vibrations. The control mechanism of dampening hand vibrations approached in this study not only supports the understanding of the role of the human CNS but also is applicable to the control of a robot in a real-world environment and the development of a human support robot.

One possible strategy is to plan and generate the desired trajectories of all the joints. In this strategy, the human CNS designs a joint angular trajectory to minimize a cost function, e.g., the hand jerk. In the field of computational neuroscience, humans plan a desired trajectory to minimize such cost functions as hand jerk [2], torque change [3], and the effect of signal-dependent noise [4] to perform a simple point-to-point reaching movement. In control engineering, such an optimization strategy manages a mechanical transfer system while suppressing the vibration [5]. Based on our earlier study that investigated the movements of human-dampening hand vibrations to keep a cup filled with water while quietly standing and constraining the usable body DOFs [6], the subjects dampened their hand vibrations while their lower limb DOFs, i.e., ankles and knees, were constrained. A platform where the subjects stood was horizontally oscillated to perturb the cup but avoid spilling water. Even though the subjects were exposed to the horizontal oscillations under a knee-constrained condition, they could dampen hand vibrations without practice. Therefore, it is unlikely that they planned and generated the desired trajectories of all of their joints by considering the changed limb dynamics and external oscillation. To dampen hand vibrations, they adopted a strategy based on such musculoskeletal system properties as equilibrium point control [7] rather than a strategy that generates a desired trajectory for all joints.

The human joint has a spring-like property that reflects a muscle property [8]. In a simple model, a joint can be driven by a pair of agonist and antagonist muscles. A change in the balance of an activity of the agonist and antagonist muscles can control the joint stiffness and an equilibrium point. If a joint torque is generated by a spring-like property, the joint torque *τ* can be estimated using a simple spring tension equation, *τ* = -*K*(*θ*–*θ*_*eq*_). The joint stiffness *K* is defined as a spring constant of the joint and increases with an increase in the magnitude of the equilibrium forces of the agonist and antagonist muscle tensions. The equilibrium point *θ*_*eq*_ is defined as the joint angles with a balance of the agonist and antagonist muscle tensions. We assume that humans utilize a joint’s spring-like dynamic property to dampen hand vibrations. Therefore, in this study, we examine a control mechanism that dampens hand vibrations by adjusting the joint stiffness or the equilibrium point. To simplify this problem, we consider a task where a horizontal oscillation is given to the subject’s shoulder, and the subject has to dampen the hand vibration from the shoulder oscillation. Then, we compare movements generated in the task using a cup filled with water and that using a cup filled with stones to examine the mechanism of dampening hand vibrations. The task using the cup filled with stones is specified to remove the effect of the inertia of the cup. We propose two schemes to dampen hand vibrations: passive and active suppression methods. The passive suppression method lowers the joint stiffness, i.e., softens the joint to passively dampen the hand vibrations. In the active suppression method, the equilibrium point is adjusted on the basis of skyhook control [9] that suppresses mechanical vibrations in engineering fields. The skyhook control assumes a virtual plane and damper in a workspace to reduce the vibrations of a controlled object in the workspace. We hypothesize that a human dampens hand vibrations by an active control of the equilibrium point, because increasing the joint stiffness is comparatively easy but decreasing it is difficult. In addition, dampening hand vibrations in a workspace is important to stabilize the cup filled with water. To test the hypothesis, our simulation experiment applies these two suppression methods and evaluates the jerk of each part of the arm and the hand trajectory amplitudes. We also conducted a measurement experiment where subjects performed the same task as in the simulation experiments. We compared the results of the simulation and measurement experiments and discussed their validity as a control model for humans.

## Materials and Methods

### Task that maintains a cup for sinusoidal oscillation

**Fig 1:**
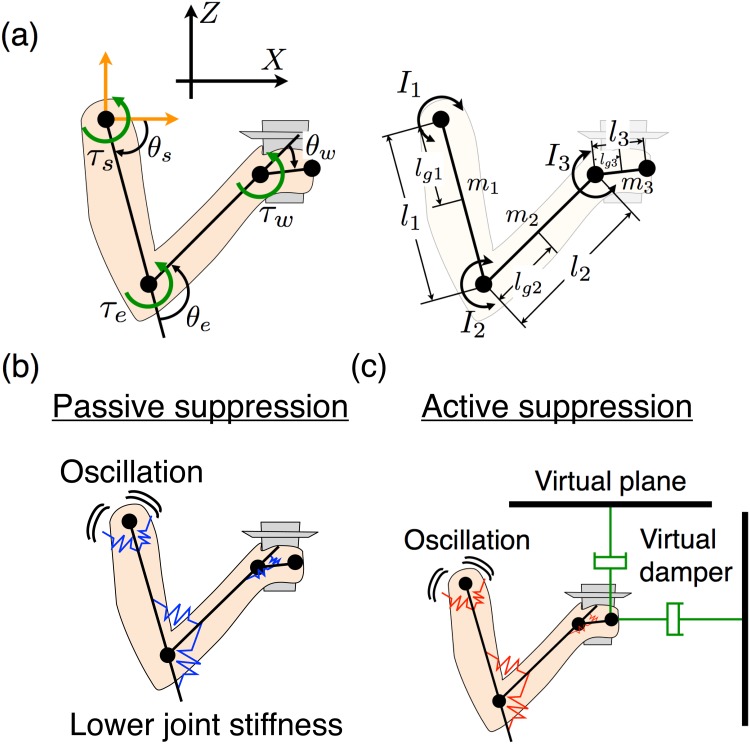
Concept of passive and active suppression methods. (a) Schematic representation of task coordinates, joint angles, and physical parameters of a three-link arm in a vertical plane. Shoulder is moved as a sinusoidal oscillation. (b) Passive suppression method. Joint stiffness is lowered to passively dampen a hand vibration generated by the shoulder’s oscillation. (c) Active suppression method. Based on skyhook control, a virtual flat plane connected to the hand by a virtual damper is considered and the equilibrium point is adjusted to actively dampen hand vibrations.

**Fig 2:**
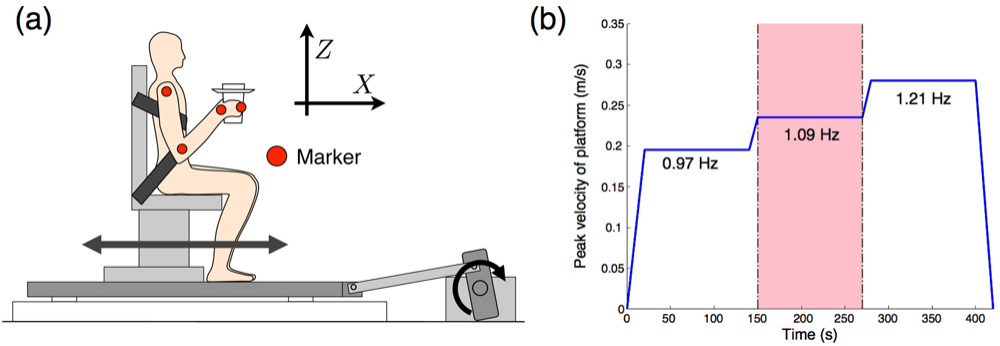
Schematic of the experimental setup. (a) Subjects held a cup filled with water or stones while sitting on a chair placed on a platform that generates a horizontal oscillation. A crank mechanism transforms motor rotation into a horizontal oscillation. Markers are placed on body segments on the right side of the arm, and these kinematic data are recorded by a three-dimensional measurement system. (b) Peak velocity of the platform oscillation. Platform is moved as a sine wave. Data in the pink area are used for the analysis.

We previously considered the vibrations generated by a subject’s natural walking [1], which are difficult to precisely address in computer simulations. To simplify this problem, we consider a dampening hand vibration task to avoid spilling water when a subject’s shoulder is sinusoidally oscillated in the anterior-posterior direction. In the simulation experiments, we assumed a three-link arm moving in the sagittal plane (Fig 1a). In the measurement experiments, we used a platform that generates a horizontal oscillation; this platform was developed in our previous study (Fig 2a) [6]. To oscillate a subject’s shoulder, a rigid chair was attached to the platform, and the subjects were fixed to chairs by using seat belts. In similar tasks for both the simulation and the measurement experiments, we gave the shoulders a sinusoidal oscillation with a 0.03-m amplitude and 1.09-Hz frequency. In both experiments, we evaluated the jerk of each part of the arm and the hand trajectory amplitudes. In our simulation experiment, we examined the following two control models (passive and active suppression models) to dampen hand vibrations and compared the results of the two experiments.

### Passive and active suppression methods

The controlled object was a three-link arm consisting of an elbow, wrist joints, and a shoulder that moves in the sagittal plane (Fig 1a). The motion equation for the three-link arm is as follows:

$$I(\theta )\ddot{\theta }+M(\theta )\ddot{X}+V(\begin{array}{cc} \theta , & \dot{\theta } \end{array})+G(\theta )+D\dot{\theta }=\tau_{in},$$ (1)

where *θ* denotes the joint angle; *X*, the shoulder position in the workspace; *I*(*θ*)*θ*+ *M*(*θ*)*Ẍ*, the inertial term; *V*, the centripetal and Coriolis term; *G*(*θ*), the gravity term; *Dθ·*, the viscous term; and *τ*_*in*_, the input torque. For this three-link arm, we consider a method to dampen hand vibrations while maintaining the arm posture, which is denoted by *τ*_*in*_.

First, we propose a method to passively dampen hand vibrations by adjusting the joint stiffness of all the joints in the three-link arm. In this method, the hardness of the joint’s spring, i.e., the joint stiffness, is lowered to reduce the hand jerk (Fig 1b). Since this method reduces the shoulder vibration’s effect on the hand by lowering the joint stiffness, we call this a passive suppression method. Considering the characteristics of human muscles, *τ*_*in*_ from the right side of Eq (1) is expressed as follows:

$$\tau_{in}=-K(\theta -\theta^{d})+G(\theta^{d}),$$ (2)

where *K* denotes the stiffness matrix; *θ* ^d^, the desired arm posture; and *G*(*θ* ^d^), the gravity compensation term. Humans seem to be able to control *K* independently of the equilibrium point. The passive suppression method maintains a constant equilibrium point with a standard value of the stiffness matrix *K* in the task using a cup without water, i.e., holding a cup filled with stones that has the same weight as a cup filled with water. In the task using a cup filled with water, *K* is lowered to reduce the high-frequency component to avoid spilling the water. Note that the desired arm posture does not change between the tasks using a cup with or without water. The input torque of Eq (2) can take any value of joint stiffness *K* to maintain the desired arm posture *θ* ^d^ by using the gravity compensation term *G*(*θ* ^d^).

Second, we propose a method to dampen hand vibrations by controlling the equilibrium point at each time step instead of controlling the joint stiffness. To achieve this result, we apply skyhook control [9], which has been used for the active suspension of a vehicle, railway [10–13], and building [14]. The skyhook theory assumes in the workspace a virtual plane, which it connects with the suppressed object by a virtual damper. A large virtual damper leads to the small vibration amplitude of the suppressed object. In other words, the connection between the virtual plane and the suppressed object by the virtual damper can dampen vibrations. To achieve movement based on skyhook control, the controller is given the feedback of the suppressed object’s state related to the virtual plane and controls an actuator as if the virtual damper actually exists. The skyhook control dampens the vibrations of the controlled object in a workspace. In the task dampening human hand vibrations while carrying a cup filled with water, stabilizing the cup in the workspace is important to avoid spilling water. Therefore, we apply the skyhook control to the active suppression method of dampening human hand vibrations. We assume that the connection between the virtual damper and a human hand can reduce the hand’s vibrations to avoid spilling the water. Fig 1c shows the control model of a human arm to dampen hand vibrations based on skyhook control. The CNS (controller) assumes that the virtual planes are connected to the hand by the virtual damper and sends a motor command to each muscle (actuator). The torque generated by the muscles based on skyhook control is as follows:

$$\tau =-J^{T}C{\dot{x}}_{ha|vp},$$ (3)

where *J* denotes the Jacobian matrix associated with the hand and angular velocities of each joint; *C*, the virtual damper; and ${\dot{x}}_{ha|vp}$, the relative hand velocity to the virtual plane. The virtual viscous force acting on hand $C{\dot{x}}_{ha|vp}$ is transformed into the joint torque using a transposed Jacobian matrix *J* ^*T*^. However, the desired arm posture cannot be maintained just by using the virtual damper’s force. Thus, a control is also required to maintain the desired arm posture, which we maintain by using the spring tension’s equilibrium (Eq (2)) with the input torque *τ*_*in*_:

$$\tau_{in}=-K(\theta -\theta^{d})+G(\theta^{d})-J^{T}C{\dot{x}}_{ha|vp}$$ (4)

When the cup is filled with stones, we hypothesize that no virtual damper is required, i.e., *C* = diag (0, 0), because the torque term remains and maintains the desired arm posture. In contrast, when the cup is filled with water, the hand vibrations are dampened with the virtual damper *C*.

### Simulation experiment

We conducted simulation experiments in which the hand vibrations from the sinusoidal oscillations of the shoulder were dampened by using passive and active suppression methods. The physical parameters of a three-link arm were calculated using anthropometric data [15]. The calculated values (Table 1) assumed a height of 172.1 cm and a weight of 63.8 kg. The mass of wrist *m*_3_ included the cup’s weight (0.39 kg, the same as that for the cup filled with water). The value of the viscous matrix of joints *D* is given in Table 2. The shoulder’s oscillation is given by *Ẍ* in Eq (1), which is derived from the 0.03-m amplitude and 1.09-Hz frequency oscillation explained in the *Task that maintains a cup for sinusoidal oscillation* section. The sampling duration was 0.01 s (100 Hz). We used the fourth-order Runge-Kutta method for the integration of the arm’s motion equation. The simulation was executed for 100 s by using both the passive and active suppression methods. We evaluated the jerk of each part of the arm and the hand trajectory amplitude in this simulation experiment. To calculate these values, we used data from 30 to 90 s and regarded them as the steady-state data.

**Table 1:** Arm parameters.

	Link 1	Link 2	Link 3
*m*_i_ kg	1.79	1.02	0.77
*l*_*i*_ m	0.24	0.22	0.17
*l*_*gi*_ m	0.10	0.09	0.08
*I*_*i*_ kgm^2^	3.00 × 10^-2^	1.34 × 10^-2^	0.73 × 10^-2^

*m*_i_, *l*_*i*_, *l*_*gi*_, and *I*_*i*_ correspond to the physical parameters shown in Fig 2a (*i* = 1, 2, 3).

**Table 2:** Viscous matrix *D* in simulation.

Joint viscosity *D* Nms/rad			
Body parts	Shoulder	Elbow	Wrist
Shoulder	1.5	0.5	0
Elbow	0.5	1.0	0
Wrist	0	0	0.4

Diagonal elements indicate joint viscosities of monoarticular muscles, and off-diagonal elements indicate those of biarticular muscles. Values of zero indicate no biarticular muscles between shoulder and wrist and between elbow and wrist.

In the passive suppression method, the value of the basic stiffness matrix *K* is given in Table 3 when the cup is filled with stones. The value of *K* is determined on the basis of the empirically estimated value of the joint stiffness during the reaching movements in the horizontal plane [16]. We examined the effect of stiffness *K* reduced by 0.1 times and 0.01 times from a previous value [16] and null stiffness and evaluated the jerk of each part of the arm and the hand trajectory amplitudes. The desired arm posture *θ* ^d^ is given by the mean measurement data of a typical subject in the task using a cup filled with water (explained in the next section).

**Table 3:** Standard stiffness matrix *K* in simulation.

Joint stiffness *K* Nm/rad			
Body parts	Shoulder	Elbow	Wrist
Shoulder	20	5	0
Elbow	5	10	0
Wrist	0	0	5

Diagonal elements indicate joint stiffness of monoarticular muscles, and off-diagonal elements indicate those of biarticular muscles. Values of zero indicate no biarticular muscles between shoulder and wrist and between elbow and wrist.

In the active suppression method, the value of virtual damper *C* is given by diag (0, 0) when the cup is filled with stones. When it is filled with water, the value of the virtual damper is given as follows using the results of measurement experiment: *C* = diag (20, 20), *C* = diag (40, 40), and *C* = diag (60, 60) Ns/m. The value of joint stiffness *K* (Table 3) was also used to generate torque to attract the desired posture (Eq (4)).

### Measurement experiment

#### Subjects

Eight healthy right-handed males participated in the experiments. Their average age was 23.0 (21–27 years), their average height was 172.1 cm (168–182 cm), and their average weight was 63.8 kg (53–70 kg). The experiments were approved by the Nagoya University Ethical Review Board. All subjects received explanations about the experimental procedure and provided written informed consent.

#### Data collection

We used a platform that generates horizontal oscillations to sinusoidally oscillate a subject’s shoulder, as shown in Fig 2a (R Techs Inc., Osaka, Japan). A crank mechanism transforms a motor rotation into a horizontal oscillation. We used a three-dimensional position measurement system (Optotrak Certus, Northern Digital Inc., Ontario, Canada) to record the kinematics data at 100 Hz. 7-mm diameter infrared-ray markers were placed on the subject’s four arm segments: the center of the gyration of the shoulder, elbow, and wrist, and the root of the digitus medius (Fig 2a). Subjects sat on a rigid chair and wore a seat belt to fix their trunks.

#### Procedure

Subjects held a cup filled with water (WW task) or with stones (WS task) in their right hand. The oscillation amplitude was 0.03 m, and the peak velocity is shown in Fig 2b. The WW task cup was filled with water to about 0.01 m from the brim. The subjects kept their right arm parallel to the sagittal plane and looked into the cup in both tasks. In the WW task, they held the cup without spilling any water, and in the WS task, they did not largely change their arm posture from that in the WW task. They practiced the WW task once before the experiment began.

#### Data analysis

To calculate the average value of a jerk of each part of the arm and the mean hand trajectory in one cycle, all the kinematics data were divided by a cycle of platform oscillation. The position data were filtered with a second-order Butterworth low-pass filter with a 5-Hz cut-off frequency. We obtained the jerk of each part of the arm by calculating the third differential of the measured position. The differentiated data were also low-pass filtered. We used 2-min data with a 1.09-Hz frequency of the oscillation indicated as an interval shown in pink in Fig 2b, because all the subjects spilled the water at the highest oscillation frequency. The mean sum of the squared jerk of each part of the arm, the mean amplitude of the hand trajectory, and the standard deviations across all subjects were calculated using the mean data of each subject (DOFs = 7).

#### Statistical analysis

To test the hypothesis that humans actively control the equilibrium point to dampen hand vibrations, we compared the results of the simulation and measurement experiments. In particular, we examined whether the active suppression method used in the simulation experiments could reproduce the values of the jerk of each part of the arm and the hand trajectory amplitudes under the WW and WS tasks conditions in the measurement experiments. We evaluated the difference in the jerks and hand amplitudes depending on the WW and WS task conditions in the measurement experiments by using statistical analysis. We performed the following statistical analysis to compare the values of the jerk of each part of the arm in both the WW and the WS tasks. We performed a two-way repeated-measures analysis of variance (ANOVA) to examine the effects of the cup condition (WW and WS tasks) and the arm parts (shoulder, elbow, wrist, and hand) with respect to the sum of the squared jerks. The Tukey–Kramer method was used for post-hoc tests (*α* = 0.05). We performed a *t*-test (*α* = 0.05) to examine the differences between the hand trajectory amplitudes in the WW and the WS tasks.

## Results

### Results of simulation experiments

**Fig 3:**
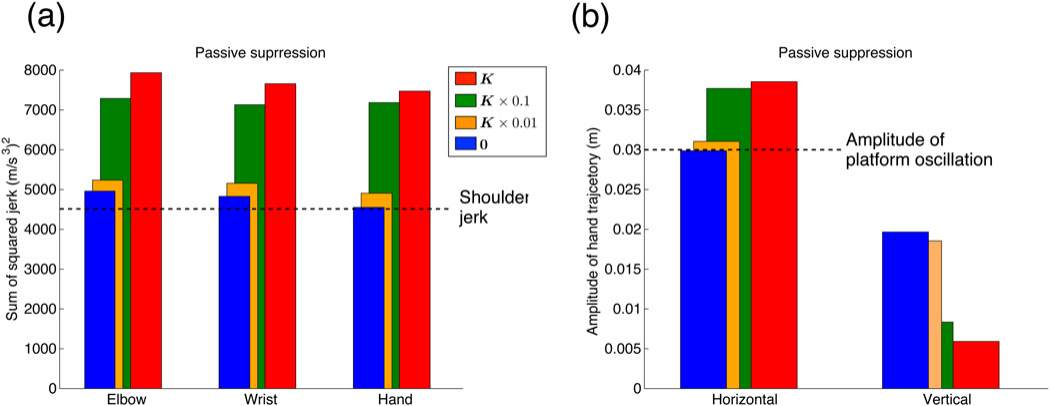
Dampening hand vibration simulated through passive suppression method. (a) Sum of the squared jerk of each part of the arm. The vertical axis denotes the sum of the squared jerk, and the horizontal axis denotes the arm part. Shoulder jerk is equivalent to the platform oscillation jerk in all conditions. Red bars indicate the values of joint stiffness *K* in the WS task, and the green, yellow, and blue bars indicate *K* × 0.1, *K* × 0.01, and 0 in the WW task, respectively. (b) Amplitude of hand trajectory. The vertical axis denotes the amplitude of the hand trajectory, and the horizontal axis denotes the amplitude direction (horizontal and vertical). Each color bar corresponds to the same conditions as in Fig 3a. Specific values of the sum of the squared jerk and the amplitude of the hand trajectory are listed in S1 Table.

Fig 3 shows the simulation results of the passive suppression method. Fig 3a and 3b show the sum of the squared jerks of each part of the arm and the hand trajectory amplitudes. When the joint stiffness values (Table 3) were based on the WS task, the hand, wrist, and elbow jerks were larger than the shoulder jerk (Fig 3a), and the hand trajectory amplitudes in the horizontal direction were larger than the oscillation amplitude of the platform (0.03 m, Fig 3b). When the joint stiffness values decreased in the WW task, the hand, wrist, and elbow jerks were reduced. These results indicate that a relatively low joint stiffness passively dampened the hand vibration due to the shoulder oscillation. However, the jerk of each part of the arm did not decrease as much as the jerk of the shoulder, irrespective of the relatively low joint stiffness values. The hand trajectory amplitude in the horizontal direction decreased, but in the vertical direction, it increased, depending on the lower joint stiffness. The hand trajectory amplitude in the horizontal direction was comparable with the platform’s oscillation amplitude (0.03 m).

**Fig 4:**
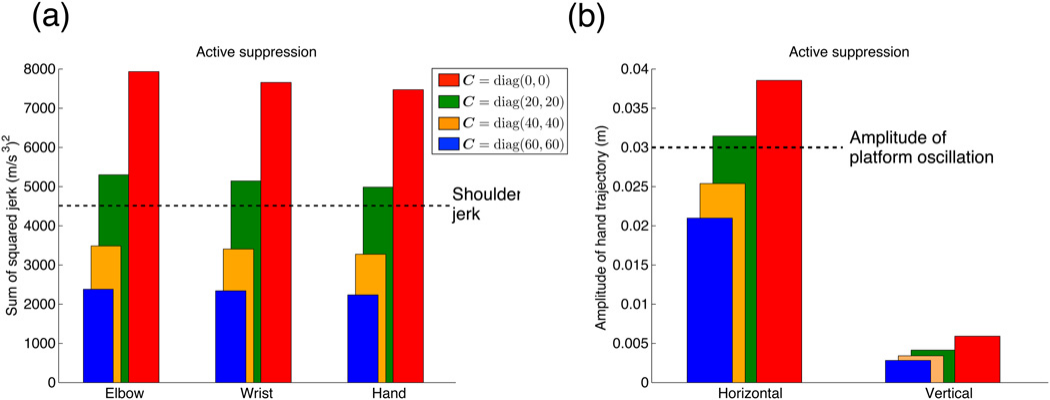
Dampening hand vibration simulated through active suppression method. (a) Sum of the squared jerk of each part of the arm. The vertical axis denotes the sum of the squared jerk, and the horizontal axis denotes the arm part. The shoulder jerk is equivalent to the platform oscillation jerk in all conditions. Red bars indicate *C* = diag (0, 0) in the WS task, and the green, yellow, and blue bars indicate *C* = diag (20, 20), *C* = diag (40, 40), and *C* = diag (60, 60) in the WW task, respectively. (b) Amplitude of the hand trajectory. The vertical axis denotes the amplitude of the hand trajectory, and the horizontal axis denotes the amplitude direction (horizontal and vertical). Each color bar corresponds to the same conditions as in Fig 4a. Specific values of the sum of the squared jerk and the amplitude of the hand trajectory are listed in S2 Table.

Fig 4 shows the simulation results of the active suppression method. Fig 4a and 4b indicate the sum of the squared jerks of each part of the arm and the hand trajectory amplitudes. The active suppression method with *C* = diag (0, 0) was equivalent to the passive suppression method (red bars in Fig 3). Therefore, the hand, wrist, and elbow jerks were larger than the shoulder jerk, and the hand trajectory amplitude in the horizontal direction was larger than the platform oscillation. When the value of *C* increased in the WW task, the hand, wrist, and elbow jerks were smaller than the shoulder jerk, and also smaller than the jerks when *C* = diag (0, 0). These results indicate that skyhook control actively dampened the hand vibration caused by the shoulder’s oscillation. Both hand trajectory amplitudes in the horizontal and vertical directions decreased with an increase in the value of virtual damper *C*. Moreover, when *C* = diag (40, 40) and *C* = diag (60, 60), the hand trajectory amplitude in the horizontal direction was smaller than the platform’s oscillation amplitude (0.03 m).

### Results of measurement experiments

**Fig 5:**
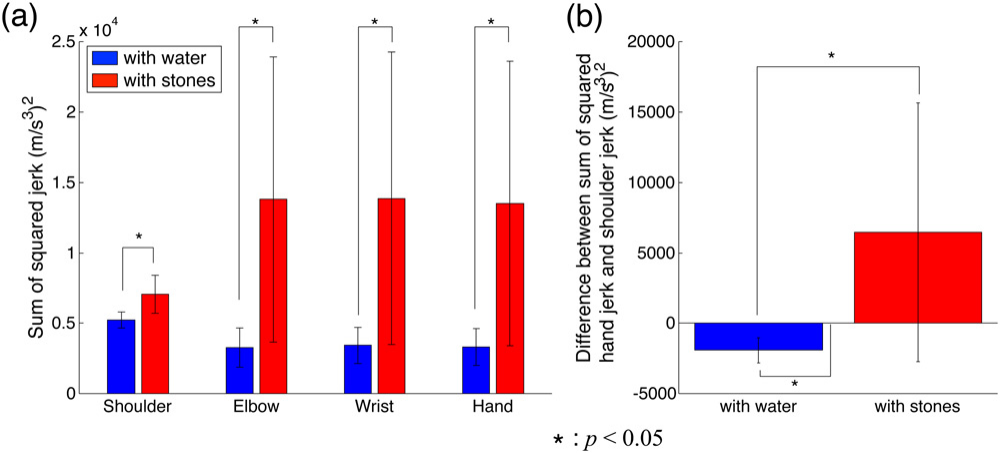
Sum of squared jerk of all subjects. (a) Sum of the squared jerk of each part of the arm across all subjects. The vertical axis denotes the sum of the squared jerk, and the horizontal axis denotes the arm part. Blue and red bars indicate the jerk in the WW and WS tasks. Asterisk denotes a significant difference. (b) Difference between the sum of the squared hand and the shoulder jerks across all subjects (hand jerk—shoulder jerk). Specific values of the sum of the squared jerk are listed in S3 Table.

Fig 5a shows the sum of the squared jerks of each part of the arm of all subjects. The blue and red bars indicate the jerk in the WW and WS tasks. The jerk of each part of the arm in the WW task was significantly smaller than that in the WS task, which indicates that the subjects could dampen the hand vibration resulting from the sinusoidal oscillation just as well as they could dampen their hand vibrations when walking [1]. Although the subjects were strapped to rigid chairs by seat belts, the shoulder jerk was slightly different between the WW and the WS tasks. The statistical analysis supported the above results. The two-way ANOVA indicated a significant difference in the jerk between the WW and the WS tasks (*F*(1, 56) = 23.81, *P*<0.05), but no significant difference was found between the arm parts (*F*(3, 56) = 0.51, *P* = 0.68) or interaction (*F*(3, 56) = 1.6, *P* = 0.19). The *t*-test showed a significant difference in the jerk of each part of the arm between the WW and the WS tasks (shoulder: *t*(7) = 0.013, *α*<0.05, elbow: *t*(7) = 0.035, *α*<0.05, wrist: *t*(7) = 0.037, *α*<0.05, and hand: *t*(7) = 0.037, *α*<0.05). Moreover, the mean elbow, wrist, and hand jerks were smaller than the shoulder jerk in the WW task and larger than the shoulder jerk in the WS task. To focus on the relationship between the shoulder and hand jerks, Fig 5b shows the difference between the sum of the squared hand and shoulder jerks. The difference in the WW task was significantly smaller than 0 (*t*(7) = 0.001, *α*<0.05), which implied that the hand jerk was significantly smaller than the shoulder jerk in the WW task. The difference in the WS task was significantly larger than that in the WW task (*t*(7) = 0.049, *α*<0.05), but not significantly larger than 0 (*t*(7) = 0.105).

**Fig 6:**
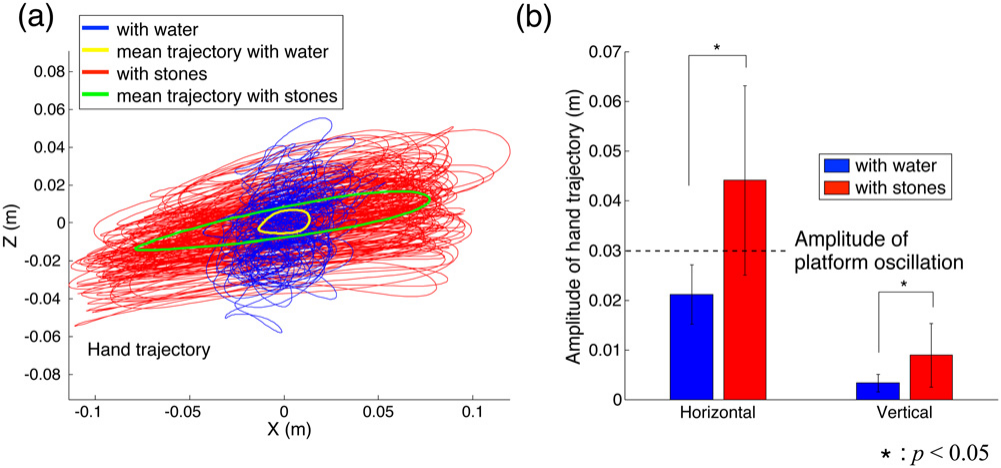
Hand trajectory. (a) Typical subject. Origin is the mean hand position. Blue and red trajectories indicate the hand trajectory for 2 min in the WW and WS tasks. Yellow and green trajectories denote the mean hand trajectory in one cycle in the WW and WS tasks. (b) Amplitude of the mean hand trajectory in one cycle of oscillation of all subjects. The vertical axis denotes the amplitude of the hand trajectory, and the horizontal axis denotes the amplitude direction (horizontal and vertical). Blue and red bars indicate the hand trajectory amplitude in the WW and WS tasks. Asterisk denotes a significant difference. Specific values of the amplitude of the hand trajectory are listed in S4 Table.

Fig 6a shows the hand trajectory of a typical subject. The origin is the mean hand position. The mean amplitude in both horizontal and vertical directions in the WW task was smaller than that in the WS task. The entire hand trajectory shows that its variation in the vertical direction was comparable between the WW and the WS tasks. To statistically analyze the hand trajectory, in Fig 6b, we show its mean amplitude in one cycle of oscillation across all subjects. In both horizontal and vertical directions, the hand trajectory amplitude in the WW task was significantly smaller than that in the WS task (horizontal: *t*(7) = 0.023, *α*<0.05 and vertical: *t*(7) = 0.045, *α*<0.05). In the WW task, the horizontal average amplitude was significantly smaller than the platform’s oscillation amplitude (0.03 m, *t*(7) = 0.006, *α*<0.05), but in the WS task, it was not significantly larger (*t*(7) = 0.089).

The results of the measurement experiments showed that the hand jerk in the WW task was smaller than the shoulder jerk, the hand jerk in the WS task was larger than the shoulder jerk, and the amplitude of the hand trajectories in both horizontal and vertical directions in the WW task were smaller than that in the WS task. These results were accurately reproduced by the active suppression method in the simulation experiments. Therefore, these results support our hypothesis that a human actively controls the equilibrium point to dampen hand vibrations in order to avoid spilling water.

## Discussion

The present study investigated the human control mechanism of dampening hand vibrations, i.e., reducing hand jerks. To simplify the problem, we considered a task where subjects dampened the hand vibrations generated by the sinusoidal oscillations of their shoulder. We proposed two control models for dampening hand vibrations: a passive suppression method that lowers the joint stiffness, and an active suppression method that controls the equilibrium point on the basis of an assumed connection between the hand and a virtual plane in the workspace by a virtual damper. Then, we hypothesized that humans actively control the equilibrium point to dampen hand vibrations in order to avoid spilling water. In the simulation experiments, we used both methods to dampen hand vibrations. To compare the results of the simulation experiments, we conducted measurement experiments for the same tasks as those tested in the simulation experiments and found that the jerk of each part of the arm in the WW task was smaller than the shoulder jerk and that in the WS task was larger than the shoulder jerk (Fig 5). For the shoulder’s sinusoidal oscillation, the subjects could reduce their hand jerk as well as dampen their hand vibrations generated during walking as examined in our previous study [1]. The hand trajectory amplitudes in both horizontal and vertical directions were significantly smaller in the WW task than those in the WS task (Fig 6). The results of the measurement experiments were well reproduced by the active suppression method based on skyhook control (Fig 4). Thus, these results supported our hypothesis. The passive suppression method also reduced the hand jerk by lowering the joint stiffness but failed to reduce it below the shoulder jerk value (Fig 3). In the WW task, subjects had to reduce the hand jerk more than the shoulder jerk in order to avoid spilling the water. Because the hand velocity was dampened relative to the external workspace, only the active suppression method reproduced the human movements in dampening the hand vibration. On the other hand, the passive suppression method can be interpreted as only using the information of the joint space because it just controls the joint stiffness. These results suggest that the human dampening of hand vibrations proceeds by controlling the equilibrium point utilizing the information of not only the internal joint space (passive method) but also the external workspace (active method).

In this study, we considered horizontal sinusoidal oscillations instead of vibrations generated during walking, which were addressed in our earlier study [1]. Considering a simple model of a cup filled with water, we found that a change in the horizontal acceleration affected the tilt of a liquid surface, but a change in the vertical acceleration did not do so. In this study, the hand jerk was smaller than the shoulder jerk in the WW task with a horizontal sinusoidal oscillation (Fig 5a). Similarly, the jerk of each part of the arm gradually dampened from hip to hand when the subjects walked while holding a cup with water without spilling it. Thus, it is possible that humans also adopt an active suppression method based on skyhook control when they walk holding a cup filled with water without spilling it.

We observed a slight difference in the shoulder jerk that was caused by the failure of the strict trunk and shoulder fixation by a seat belt (Fig 5a). In the WW task, the subjects dampened the shoulder vibration resulting from the platform oscillation by the movement of trunk to dampen hand vibrations, which was the same strategy as that observed in our previous study [1]. This suggests that the trunk also plays an important role in dampening hand vibrations. This was also suggested in earlier studies that argued that the trunk dampened the head vibrations generated by walking [17–18], and our previous study showed that hand vibrations could be dampened irrespective of the lower limb constraints [6]. In a future work, we would like to investigate, in detail, the role of the trunk movement in dampening hand vibrations.

In the measurement experiment’s WS task, we observed large variances of the jerk of each part of the arm and the hand trajectory amplitudes across all subjects because the subjects were only instructed to hold the cup filled with stones without a large change in the arm posture as compared to that in the WW task. Therefore, the large variance in the WS task might result from various joint stiffness values across the subjects. To achieve the WS task, the subjects could slightly reduce the hand jerk to stably hold the cup. Based on the results of our simulation experiments with the passive suppression method, the change in the value of joint stiffness *K* changed the values of the jerk of each part of the arm and the amplitude of the hand trajectory (Fig 3). Perhaps, the DOFs of this task led to a large variance across all subjects.

This study considered a WS task where the subjects only held a cup filled with stones. To achieve this task, we addressed the spring tension acting on the displacement from the desired arm posture (Eq (3)) in the simulation experiments. The task could also be achieved through a strategy where the inverse statics is calculated. In a static state, a strategy calculating the desired torque is equivalent to one considering the equilibrium of spring tension. Humans can acquire internal models related to the force exerted by gravity [19]. The study of human movement in the gravity’s direction suggests that humans plan movements by utilizing information about gravity [20–25]. In movements when they are holding objects, humans can also adopt a strategy that calculates the required torque to maintain the desired arm posture and continue to generate it. Such a strategy is often used in robotics to compensate the force exerted by gravity and plan the movement trajectory [26]. A strategy that generates the desired torque corresponds to *K* = 0 in the passive suppression method. Here, the hand jerk was not larger than the shoulder jerk, and the hand trajectory amplitude was not larger than the platform’s oscillation amplitude (blue bars in Fig 3). On the other hand, the WS task results of the measurement experiments revealed that the hand jerk was larger than the shoulder jerk (Fig 5) and the amplitude of the hand trajectory was larger than the platform oscillation (Fig 6), as reproduced by the spring tension’s equilibrium (Eq (2)). These results suggest that humans achieve the simple task of holding an object by maintaining the equilibrium point of the agonist and antagonist muscles in order to maintain the desired arm posture.

### Validity of active suppression method as a control model in humans

In our experiments, the human movements involved in dampening hand vibrations due to the sinusoidal oscillation of the shoulder were accurately reproduced by our active suppression method. However, the equilibrium point is moved, assuming a connection between the hand and a virtual plane, by a virtual damper. According to Eq (4) and the spring tension equation (τ = -*K*(θ- θ_eq_)), the equilibrium point θ_eq_ is updated as follows in the active suppression method:

$$\theta_{eq}=\theta^{d}-K^{-1}G(\theta^{d})-K^{-1}J^{T}C{\dot{x}}_{ha|vp}$$ (5)

The first term on the right-hand side *θ* ^d^-*K* ^-1^ *G*(*θ* ^d^) is constant, and the equilibrium point maintains the desired arm posture. In the WS task, since no virtual damper is required (*C* = diag (0, 0)), the desired equilibrium point is constant to maintain the desired arm posture. In the WW task, the virtual damper’s value needs to be appropriately determined. To use skyhook control, the following are required: the inverse matrix of joint stiffness *K*, Jacobian matrix *J*, and the relative velocity of the hand to the virtual plane x_ha|vp_. Except for the relative hand velocity, all are internal parameters of the human body. How do humans perceive relative hand velocity? One possibility is visual information. The relative hand velocity was estimated from the relative position between a hand and the background or the shaking of the liquid’s surface. However, since the platform’s oscillation amplitude was comparatively small (0.03 m), it was difficult to perceive the relative hand velocity from a comparison with the background. In daily life, humans can carry a cup filled with water without constant concentration. Thus, visual information plays an auxiliary role in tasks for dampening human hand vibrations. Another possibility is vestibular sensory information. The relative velocity of the hand to the virtual plane can be represented using the relative velocity of the head to the virtual plane:

$${\dot{x}}_{ha|vp}=J_{ha|he}\dot{\theta }+{\dot{x}}_{he|vp},$$ (6)

where *J*_ha|he_ denotes the Jacobian matrix associating the hand and head velocities and x_he|vp_ represents the relative velocity of the head to the virtual plane. The head velocity can be perceived by vestibular sensory information from the head. Therefore, an active suppression method based on skyhook control can dampen hand vibrations without any visual information of the hand. CNS dampens hand vibrations by using vestibular sensory information, information corresponding to the Jacobian matrix, and the somatosensory feedback information of the joint angle and the angular velocity.

Humans can voluntarily and predictively adjust impedance parameters such as joint stiffness and viscosity [27–28], particularly the co-contractions of the flexor and extensor muscles that increase joint stiffness. Such an increase in joint stiffness has been reported during movements in a force field [29] and in a task requiring high accuracy [30–31]. Even though it is easy to increase the joint stiffness by the co-contraction of agonist and antagonist muscles, decreasing it is difficult. The viscous elastic properties of the muscles interfere with the lowering of the joint stiffness, as in the passive suppression method used in our simulation experiments. Therefore, an active suppression method, which dampens the hand vibrations generated by controlling the equilibrium point, has validity. In the next step of this study, we have to empirically quantify joint stiffness while dampening the hand vibrations. Previous studies estimated the impedance parameters of human joints with a small perturbation using a manipulandum that can perturb a subject’s hands by the force of a motor [8], [16], [32–35] and with electromyogram (EMG) data [36]. However, the estimation of impedance parameters during movements is difficult. To estimate them while dampening hand vibrations, the manipulandum requires a platform that generates oscillations and slightly perturbs the subject’s hand at a high sampling rate. These developments and experiments are important future work.

## Conclusions

We investigated the control mechanism for dampening hand vibrations. We proposed two methods to suppress hand vibrations based on the spring-like property of the human joint: a passive suppression method that lowers joint stiffness by only referring to internal body coordinates and an active suppression method that adjusts an equilibrium point by referring to both internal body coordinates and external workspace coordinates based on skyhook control. In the simulation experiments, we applied these two methods to dampen hand vibrations generated by the shoulder’s horizontal oscillations. We also conducted measurement experiments wherein a subject’s shoulder was sinusoidally oscillated by a platform that generated horizontal oscillations. The results of the measurement experiments showed that the jerk of each part of the arm was smaller than the shoulder jerk in a task using a cup filled with water and larger than the shoulder jerk in a task using a cup filled with stones. In addition, the hand trajectory amplitudes in both horizontal and vertical directions were smaller in a task that used a cup filled with water than those in the task using a cup filled with stones. The results of the measurement experiments were well reproduced by the active suppression method by using information of both the internal joint space and external workspace. Meanwhile, the passive suppression method that uses the only information of the joint space could not reproduce the results of the jerk of each part of the arm and the amplitude of the hand trajectory in the measurement experiments. We conclude that the human controls the equilibrium point to dampen hand vibrations by using not only the information of the joint space but also information of the workspace. Empirically quantifying human joint stiffness during a task that dampens hand vibrations will be a part of future work.

## Supporting Information

S1 Table Specific values of the sum of the squared jerk and the amplitude of the hand trajectory shown in Fig 3. (DOCX)

S2 Table Specific values of the sum of the squared jerk and the amplitude of the hand trajectory shown in Fig 4. (DOCX)

S3 Table Specific values of the sum of the squared jerk of all subjects shown in Fig 5. (DOCX)

S4 Table Specific values of the amplitude of the hand trajectory of all subjects shown in Fig 6b. (DOCX)
