# Supplementary material for: Control Model for Dampening Hand Vibrations Using Information of Internal and External Coordinates
Source: PLoS One. 2015 Apr 13;10(4):e0125464. doi: 10.1371/journal.pone.0125464 (PMC4395142; doi:10.1371/journal.pone.0125464)
Supplement: S1 Table — (DOCX) [file pone.0125464.s001.docx]

Fig. 3a

|  | Elbow | Wrist | Hand |
| --- | --- | --- | --- |
| ***K*** | 8050.5 | 7759.0 | 7570.0 |
| ***K***$\times$0.1 | 7340.8 | 7182.3 | 7246.7 |
| ***K***$\times$0.01 | 5218.9 | 5143.8 | 4883.7 |
| 0 | 4940.0 | 4814.1 | 4523.1 |

Shoulder jerk: 4509.8

Fig. 3b

|  | Horizontal | Vertical |
| --- | --- | --- |
| ***K*** | 0.0388 | 0.0062 |
| ***K***$\times$0.1 | 0.0378 | 0.0085 |
| ***K***$\times$0.01 | 0.0310 | 0.0188 |
| 0 | 0.0298 | 0.0199 |
