# Supplementary material for: Control Model for Dampening Hand Vibrations Using Information of Internal and External Coordinates
Source: PLoS One. 2015 Apr 13;10(4):e0125464. doi: 10.1371/journal.pone.0125464 (PMC4395142; doi:10.1371/journal.pone.0125464)
Supplement: S2 Table — (DOCX) [file pone.0125464.s002.docx]

Fig. 4a

|  | Elbow | Wrist | Hand |
| --- | --- | --- | --- |
| ***C*** = diag(0, 0) | 8050.5 | 7759.0 | 7570.0 |
| ***C*** = diag(20, 20) | 5297.4 | 5139.4 | 4981.2 |
| ***C*** = diag(40, 40) | 3450.2 | 3368.2 | 3236.0 |
| ***C*** = diag(60, 60) | 2339.6 | 2299.3 | 2189.9 |

Shoulder jerk: 4509.8

Fig. 4b

|  | Horizontal | Vertical |
| --- | --- | --- |
| ***C*** = diag(0, 0) | 0.0388 | 0.0062 |
| ***C*** = diag(20, 20) | 0.0314 | 0.0042 |
| ***C*** = diag(40, 40) | 0.0253 | 0.0034 |
| ***C*** = diag(60, 60) | 0.0208 | 0.0028 |
