# Supplementary material for: Control Model for Dampening Hand Vibrations Using Information of Internal and External Coordinates
Source: PLoS One. 2015 Apr 13;10(4):e0125464. doi: 10.1371/journal.pone.0125464 (PMC4395142; doi:10.1371/journal.pone.0125464)
Supplement: S3 Table — (DOCX) [file pone.0125464.s003.docx]

Fig. 5 a

|  | Shoulder | | Elbow | |
| --- | --- | --- | --- | --- |
|  | WW | WS | WW | WS |
| Sub. A | 0.558$\times$10^4^ | 0.613$\times$10^4^ | 0.292$\times$10^4^ | 0.968$\times$10^4^ |
| Sub. B | 0.438$\times$10^4^ | 0.827$\times$10^4^ | 0.191$\times$10^4^ | 3.471$\times$10^4^ |
| Sub. C | 0.629$\times$10^4^ | 0.648$\times$10^4^ | 0.655$\times$10^4^ | 0.729$\times$10^4^ |
| Sub. D | 0.522$\times$10^4^ | 0.733$\times$10^4^ | 0.324$\times$10^4^ | 1.030$\times$10^4^ |
| Sub. E | 0.453$\times$10^4^ | 0.568$\times$10^4^ | 0.163$\times$10^4^ | 0.303$\times$10^4^ |
| Sub. F | 0.506$\times$10^4^ | 0.523$\times$10^4^ | 0.378$\times$10^4^ | 0.482$\times$10^4^ |
| Sub. G | $0.524 \times$10^4^ | 0.787$\times$10^4^ | $0.274 \times$10^4^ | 2.429$\times$10^4^ |
| Sub. H | 0.549$\times$10^4^ | 0.947$\times$10^4^ | 0.344$\times$10^4^ | 1.618$\times$10^4^ |
| Average | 0.522$\times$10^4^ | 0.706$\times$10^4^ | 0.328$\times$10^4^ | 1.379$\times$10^4^ |
| SD | 0.060$\times$10^4^ | 0.144$\times$10^4^ | 0.151$\times$10^4^ | 1.083$\times$10^4^ |

|  | Wrist | | Hand | |
| --- | --- | --- | --- | --- |
|  | WW | WS | WW | WS |
| Sub. A | 0.279$\times$10^4^ | 0.915$\times$10^4^ | 0.263$\times$10^4^ | 0.860$\times$10^4^ |
| Sub. B | 0.250$\times$10^4^ | 3.585$\times$10^4^ | 0.237$\times$10^4^ | 3.502$\times$10^4^ |
| Sub. C | 0.624$\times$10^4^ | 0.734$\times$10^4^ | 0.619$\times$10^4^ | 0.726$\times$10^4^ |
| Sub. D | 0.319$\times$10^4^ | 1.040$\times$10^4^ | 0.302$\times$10^4^ | 1.040$\times$10^4^ |
| Sub. E | 0.170$\times$10^4^ | 0.290$\times$10^4^ | 0.164$\times$10^4^ | 0.281$\times$10^4^ |
| Sub. F | 0.404$\times$10^4^ | 0.513$\times$10^4^ | 0.400$\times$10^4^ | 0.504$\times$10^4^ |
| Sub. G | 0.306$\times$10^4^ | 2.378$\times$10^4^ | 0.294$\times$10^4^ | 2.276$\times$10^4^ |
| Sub. H | 0.394$\times$10^4^ | 1.639$\times$10^4^ | 0.378$\times$10^4^ | 1.625$\times$10^4^ |
| Average | 0.343$\times$10^4^ | 1.387$\times$10^4^ | 0.332$\times$10^4^ | 1.352$\times$10^4^ |
| SD | 0.136$\times$10^4^ | 1.109$\times$10^4^ | 0.138$\times$10^4^ | 1.079$\times$10^4^ |

Fig 5b

|  | WW | WS |
| --- | --- | --- |
| Sub. A | $-$2949.9 | 2470.6 |
| Sub. B | $-$2011.7 | 26743.5 |
| Sub. C | $-$100.0 | 779.9 |
| Sub. D | $-$2196.6 | 3078.0 |
| Sub. E | $-$2893.1 | $-$2875.8 |
| Sub. F | $-$1063.5 | $-$186.3 |
| Sub. G | $-$2305.7 | 14892.1 |
| Sub. H | $-$1704.3 | 6779.4 |
| Average | $-$1903.1 | 6460.2 |
| SD | 951.0 | 9809.4 |
