# Supplementary material for: Control Model for Dampening Hand Vibrations Using Information of Internal and External Coordinates
Source: PLoS One. 2015 Apr 13;10(4):e0125464. doi: 10.1371/journal.pone.0125464 (PMC4395142; doi:10.1371/journal.pone.0125464)
Supplement: S4 Table — (DOCX) [file pone.0125464.s004.docx]

Fig. 6b

|  | Horizontal | | Vertical | |
| --- | --- | --- | --- | --- |
|  | WW | WS | WW | WS |
| Sub. A | 0.0188 | 0.0344 | 0.0048 | 0.0028 |
| Sub. B | 0.0135 | 0.0779 | 0.0062 | 0.0154 |
| Sub. C | 0.0320 | 0.0363 | 0.0017 | 0.0040 |
| Sub. D | 0.0226 | 0.0438 | 0.0046 | 0.0082 |
| Sub. E | 0.0128 | 0.0153 | 0.0023 | 0.0037 |
| Sub. F | 0.0258 | 0.0274 | 0.0014 | 0.0028 |
| Sub. G | 0.0200 | 0.0633 | 0.0017 | 0.0190 |
| Sub. H | 0.0244 | 0.0547 | 0.0047 | 0.0162 |
| Average | 0.0212 | 0.0442 | 0.0034 | 0.0090 |
| SD | 0.0064 | 0.0203 | 0.0018 | 0.0068 |
